# Creative Togetherness. A Joint-Methods Analysis of Collaborative Artistic Performance

**DOI:** 10.3389/fpsyg.2022.835340

**Published:** 2022-03-28

**Authors:** Vincent Gesbert, Denis Hauw, Adrian Kempf, Alison Blauth, Andrea Schiavio

**Affiliations:** ^1^Football Club Lorient, Lorient, France; ^2^Institute of Sport Sciences, Faculty of Social and Political Sciences, University of Lausanne, Lausanne, Switzerland; ^3^Center for Systematic Musicology, University of Graz, Graz, Austria; ^4^Artistic Swimming Swiss National Federation, Lausanne, Switzerland

**Keywords:** togetherness, joint performance, individuality, collectivity, sports psychology

## Abstract

In the present study, we combined first-, second-, and third-person levels of analysis to explore the *feeling of being and acting together* in the context of collaborative artistic performance. Following participation in an international competition held in Czech Republic in 2018, a team of ten artistic swimmers took part in the study. First, a self-assessment instrument was administered to rate the different aspects of togetherness emerging from their collective activity; second, interviews based on video recordings of their performance were conducted individually with all team members; and third, the performance was evaluated by external artistic swimming experts. By combining these levels of analysis in different ways, we explore how changes in togetherness and lived experience in individual behavior may shape, disrupt, and (re-)stabilize joint performance. Our findings suggest that the experience of being and acting together is transient and changing, often alternating phases of decrease and increase in felt togetherness that can be consistently recognized by swimmers and external raters.

## Introduction

Individuals displaying high-level expertise in sports and the arts usually operate in a performative niche involving multi-leveled layers of reciprocal interaction (Carron et al., 2002). For example, many skilled musicians perform in ensembles, play for an audience, learn music with and through others, and develop important relationships with the cultural norms and narratives sedimented in their social and historical environment. Similarly, athletes often rely on team effort and develop their skills through training sessions that are in most cases collaborative (e.g., with a coach, other athletes, etc.). Being together with others is therefore increasingly understood as a fundamental resource that can shape skill acquisition and creative performance across a range of individual and collective contexts (see Davids et al., 2007; Hauw, 2018; Schiavio et al., 2019). Although this dimension of being together is perhaps less apparent in instances of solitary musicking and sports performance (but see Høffding and Satne, 2019; Schiavio et al., 2020, in press), it is clearly manifested in a population of musical ensemble and sports team members, whose performances are constantly organized and carried out through a moment-to-moment participation with co-performers, team members, audience, and/or opponents.

There is a vast literature on the psychological dynamics associated with creative teamwork in sports and the performing arts, including studies focused on *group cohesion* (see e.g., Spink, 1990; Heuzé et al., 2006; Lund et al., 2014; Glowinski et al., 2016), *collective creativity* (Santos et al., 2016, 2017; Bishop, 2018), *coordination dynamics* (Keller et al., 2014; Laroche et al., 2014; Miyata et al., 2017; Himberg et al., 2018) as well as *synchrony and self-other overlap* (Lakens, 2010; Lakens and Stel, 2011; Rabinowitch and Knafo-Noam, 2015; Tunçgenç and Cohen, 2016). Group cohesion is usually defined as “a dynamic process that is reflected in the tendency for a group to stick together and remain united in the pursuit of its instrumental objectives and/or for the satisfaction of member affective needs” (Carron et al., 1998, p. 213). In this process of reciprocal collaboration, novel (e.g., expressive, behavioral) joint configurations can emerge, giving rise to creative outcomes that play out at different layers of awareness (see Walton et al., 2015; Orth et al., 2017; Kimmel et al., 2018).

Examples can be found in studies investigating how action patterns developed in response to unexpected occurrences during performance (e.g., a novel strategy displayed by the opponent team, an unanticipated subtle change in the re-creation of the musical score by a co-performer, etc.) can lead to functional and innovative (or indeed, *creative* – see Runco and Jager, 2012) modifications in behavior. Here contextual adaptations and activities are often negotiated in both local (e.g., what action can be performed individually?) and global (e.g., what collective configuration can emerge from individual behaviors?) terms. Consider how in a soccer game, for instance, a range individual and collective factors, like tiredness or a change in tactics, are highly co-dependent and can shape how teammates respond to particular contextual contingencies – an example being the modification of an existing defensive strategy (see Duarte et al., 2012). Here adaptations are thought to be continuously developed in response to a range of moment-to-moment perturbations that disrupt the stability of the joint activity (see Gesbert and Durny, 2017; van der Schyff et al., 2018; Schiavio et al., 2021).

Within such contexts, these evolving behavioral modifications have been increasingly studied in terms of *coordination dynamics* (see Kelso, 2001, 2003; Tognoli et al., 2020). The main idea is to conceive of a joint performance as a uniquely structured system based on a reciprocal interplay of biological and ecological parameters that recursively change over time (see Chow et al., 2011; Seifert et al., 2013). As such, the set of constraints and open possibilities offered by the physical and social environment in which the performance unfolds is functionally coupled with the shifting behavioral trajectories of the performers, giving rise to a distributed network of co-dependencies that sustains, transforms, and re-orients the joint performance (Chemero, 2009; Hristovski et al., 2012). The constant re-organization of this agent-environment system involves (creative) changes that play out at both macro- and micro-scales (see Demos et al., 2018; Schiavio and Benedek, 2020). Accordingly, not only do kinematics, motor plans, predictions, and outcomes exhibit visible modifications, but the subtle, personal experiences that permeate joint performances are also subject to transformations in the here-and-now. Genuinely subjective descriptions of these dynamics are notoriously difficult to obtain, and they escape the analytic approaches relying on quantitative methods. Yet, gaining a deeper understanding of the individual experiences involved in collective behaviors is of major importance for developing a more integrated view of joint activity – one that places equal emphasis on its local and global components, as well as on both experiential and behavioral dimensions (see Tanaka, 2017; Høffding, 2019).

In this study, we aimed to contribute to this line of research by exploring in greater detail the experience of being and acting together (or “togetherness”) emerging from collaborative performance (see Bourbousson and Fortes-Bourbousson, 2017). According to Himberg et al. (2018), the feeling of being and acting with others is indeed an essential part of collective performance as it facilitates the regulation of individual and collective behaviors in light of the direct, immediate experience of others (see Colombetti and Torrance, 2009; Froese and Di Paolo, 2011; He and Ravn, 2018). How do performers describe their experience of being and acting together? What role does it play in regulating and optimizing joint action? And can this sense of togetherness be perceived from the outside, for example, by an audience? To provide some preliminary answers, we report on an original study that focused on collaborative artistic activity (synchronized swimming) and adopted a “joint-methods” approach – one that combines first-, second- and third-person levels of analysis. The first-person data were generated via the administration of a self-assessment instrument, the second-person data were based on interviews (see Petitmengin, 2006), and the third-person data were obtained from independent raters who assessed the swimmers’ performance. We suggest that this methodology may provide rich understandings of the creative dynamics of skilled action during participatory activity, offering insights generated on intrapersonal and interpersonal levels. This can mutually “validate and constrain” empirical data generated via more traditional methods (Varela and Shear, 1999, p. 6).

### Rationale for the Study

Research exploring the interplay of individual and collective dynamics usually focuses on two levels of description: phenomenological and behavioral (Hauw, 2018; Gesbert and Hauw, 2019). To bring together and complement these two lines of inquiry, Seifert et al. (2016) offered a more unifying approach to study interpersonal coordination in sports based on mixed methods. This approach combines two analytical strategies: the first explores the continuities and discrepancies between behavioral and phenomenological data via side-by-side comparisons, and the second integrates theory-driven categories with tools from ecological dynamics. A good example of the first strategy is the *neurophenomenological* approach, which relies on quantitative analyses of measurable phenomena related to brains, bodies and behaviors, while “embracing the value of first-person reports of experience” (Bockelman et al., 2013; see also Varela, 1996; Lutz, 2002; Lutz et al., 2002; Petitmengin and Lachaux, 2013; Depraz and Desmidt, 2019). Concerning the second line of enquiry proposed by Seifert et al. (2016), one might consider how the recent work by Kimmel and Rogler (2018, 2019) applies theoretical resources from micro-phenomenology, cognitive ethnography, and ecological dynamics to explore qualitatively how agents carry out high-level interacting skills in the context of Aikido. In their examination of the moment-to-moment web of interactivities unfolding between performers, they found that “collective dynamics and individual affordances dialectically engender each other” (Kimmel and Rogler, 2018, p. 251), pointing to the co-specification of individuality and collectivity in embodied decision-making during emergent collaborative activity.

As the datasets in this type of study are often complex, many researchers have moved from a *mixed-methods* approach (see Anguera et al., 2017) to *joint-methods* (see e.g., Poizat et al., 2012; Sève et al., 2013; R’Kiouak et al., 2016; Hauw et al., 2017; Seifert et al., 2017; Rochat et al., 2020). The former refers to the practice of juxtaposing and/or comparing qualitative and quantitative data to cross-correlate specific aspects of experience with possible behavioral outcomes (see e.g., Vors et al., 2019), whereas the latter instead uses two domains of evidence to enrich an initial analysis based on data with a specific format. In this case, there is *a first choice that determines how additional data can be successfully integrated*. For example, Rochat et al. (2019) recently conducted a study explicitly inspired by such an approach to investigate the experiences of trail-runners interacting with five different water-carrying systems. Nine runners were equipped with a carrying system (e.g., a backpack with two front bottles on the shoulder straps; a waist pack with the bottles on the hips, etc.) and ran a 3-kilometer loop at a regular pace; at the end of each trial, they were instructed to change the water-carrying system, and they then repeated the loop with another carrying system. They repeated the loop five times, each time with a different system. For each trial, the runners were also equipped with inertial sensors to measure both their vertical oscillations and those of the five carrying systems. After the five loops, the runners were individually interviewed and asked about the different “traces” of their past activity, such as pictures and maps of the route and pictures of themselves during the transitions between trials. This confrontation was designed to help the runners access and describe their experience at the moment their activity was unfolding. More specifically, the authors first sought to document the salient aspects associated with the carrying systems during the unfolding activity at the phenomenological level in order to determine the relevant dependent variables to investigate (e.g., when the runners described disturbing system elements like the feeling of the system bouncing in an uncomfortable way). From these qualitative insights, two hypotheses emerged in relation with the behavioral data (i.e., the vertical oscillations of the runners’ hip and the backpack) characterizing low- and high-order parameters of behavior, such as the couplings between the accelerations of the runners and the backpacks.

The present study builds on these methodological insights to explore the “feeling of being and acting together” or “sense of togetherness” associated with the ability of team members to successfully coordinate with each other. The study took place during an international competition held in 2018 in Czech Republic, where members of a team of swimmers performed a free combination routine, which was then assessed at the three above-mentioned levels (1st person, 2nd person, and 3rd person). Synchronized swimming is a form of collective performance involving two, eight, or ten swimmers performing a synchronized routine of elaborate moves in the water, accompanied by music. We chose synchronized swimming because this activity demands elaborate individual and collaborative skills – for example, propelling the body through hand movements while performing upside down, achieving stability and height above the water while leaving the hands free to perform arm motions, gaining a sense of how the team is performing, and so on. Here the creative aspects of the process relate to those real-time strategies that swimmers adopt to compensate for possible problems in the exercise, thereby regaining coordination in different ways.

Among others, there are two important questions a joint-methods analysis can help answer: (i) how can changes in individual performance disrupt the unfolding dynamics of interpersonal coordination? and (ii) how can swimmers compensate for destabilizations in team performance and regain individual and collective stability? Moreover, the elision of individual behavior and ecological constraints (i.e., the music guiding performance, the particular environment in which it takes place, etc.) complements the research to date on behavioral co-adaptation and joint action. For example, Froese et al. (2014) investigated how social agents actively co-regulate their interactions in the service of joint action, but no aesthetic or creative dimension was considered. Given its individual, collaborative, and ecological complexity, synchronized swimming is an ideal candidate for investigation.

## Methods

### Participants

Ten artistic swimmers participated in this study. They were between 15 and 22 years old (*M* = 17.8; *SD* = 2.4) and had been practicing artistic swimming between 8 and 13 years at the time of the study (*M* = 9.9; *SD* = 1.7). They were informed of the study purpose and told that their participation was entirely voluntary. Before the study began, they or their families (for those under 18 years) approved, and gave written consent to a protocol agreement that described the study purposes in detail and ensured confidentiality and anonymity (i.e., swimmers were given pseudonyms). Participants were already known to the first author due to an ongoing collaboration. They were not monetarily compensated for their participation. In addition to these swimmers, five other expert swimmers, blind to the aims of the study, were recruited to perform an observational analysis of the swimmers’ performance. Their age was 28.4 on average (*SD* = 5.9). They had all been artistic swimmers at the international level and had trained between 5 and 15 years (*M* = 8; *SD* = 4). They were recruited by the fourth author, and they were not monetarily compensated. The study project was not submitted for approval to the Ethics Commission of University of Lausanne as it did not fall within the legal obligations in Switzerland. Indeed, according to Swiss law, only studies dealing with health data must be submitted to an Ethics Commission for authorization. Since this was not the case for our study, we were not required to do so. Nevertheless, the data collection respected the common ethics rules in psychology and was in accordance with the Declaration of Helsinki: the procedures for data collection and analysis were explained in detail to the participants, who gave written informed consent to participate, as did parents/guardians for those under 18 years. The athletes’ anonymity was guaranteed by an anonymous login created by each athlete and only the first researcher knew the link between the athlete and the login.

### Data Collection

Data were collected during an international competition that took place in 2018. The main focus of the study was a free combination routine performed by ten swimmers. A *free combination* is a routine that may be a compound set of solos, duets, trios and other team segments. No technical event is “prescribed” for the free combination routine, and swimmers can be quite creative by, for example, presenting higher and bigger lifts. The observed routine lasted about 4 min and 20 s and it was the first time it was performed in competition. Three types of data were collected: (i) first-person, with the swimmers’ self-assessments of their feelings of being and acting together during the routine. These were collected just after competition using Likert scales administered to each participant; (ii) second-person, concerning the swimmers’ qualitative experiences during the routine. These were collected between 1 and 5 days after the competition by individually confronting the swimmers with the video of their performance; and (iii) third-person, based on the experts’ assessments of togetherness. One week after competition, these raters evaluated the team performance and individuated units of behavioral team activity through observational analysis of the video recordings. In what follows, we provide more detailed information concerning the units of analysis and the three types of data reported above.

Before the data were collected, two independent experts (blind to the purposes of the study) observed the choreography in the last training sessions before competition and then were asked to segment the team performance (i.e., routine) into discrete collective units of behavioral activity (see Zacks and Swallow, 2007; Kurby and Zacks, 2008, for a similar approach). The video was segmented into 33 units. This segmentation allowed us to capture the shifting dynamics of being together (at first- and third-person levels) and provided the ground from which the subsequent interviews were structured. The team performance was considered a unitary system, with each component sustaining and regulating the continuous dynamical interplay between its different phases and trajectories (see van der Schyff et al., 2018). On this basis, we conceived of the system transitions as emerging units for analysis. These involved series of episodes or segments with a clearly visible beginning and end. Indications of the end of a unit might be a change in team organization, ending a figure, or moving in a new direction. Put differently, each collective unit corresponded to a behavioral shift within team performance, in a sense similar to what Himberg et al. (2018) described as sudden bifurcations from one state to another. Consider, for example, collective unit of activity 2, which is topographically and contextually represented in, respectively, Figures 1a,b.

**FIGURE 1a:**
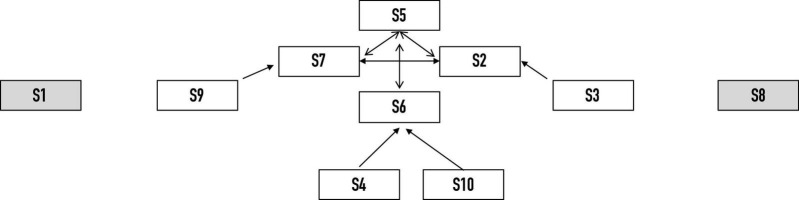
Topographic representation of collective unit of activity 2.

**FIGURE 1b:**
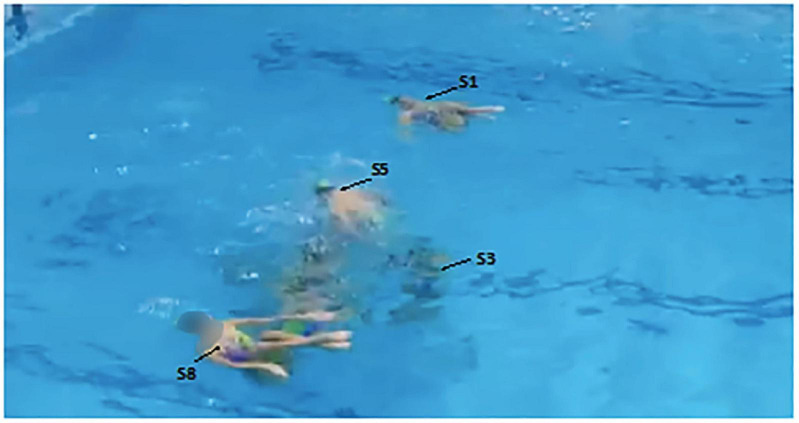
Contextual representation of The collective unit of activity 2.

Here, the position of each swimmer corresponds approximately to her position in the swimming pool during the collective unit of activity. Each swimmer was labeled S1, S2, and so on. The arrows between two swimmers meant bodily contact (e.g., bodily contact was established between S5 and S7, S2 and S6, etc.). For this collective unit, the team was split into eight and two swimmers. Indeed, S1 and S8 were expected to be aligned on both sides of the highlight produced by the eight other swimmers.

#### Self-Assessments

After competition, all swimmers were invited to assess their feelings of acting together on Likert scales. The purpose was to explore team performance from a *first-person* perspective across the 33 collective units of activity on 7-point scales ranging from: (1) “I had the feeling that we did not act together” to (7) “I had the feeling that we acted together.” The scores of all swimmers were then summed up together to obtain a team score for each unit (the maximum score for the team was 70, see Table 1).

**TABLE 1:** Illustration of first- and third-person data in relation with the swimmers’ feeling of being and acting together and the experts’ assessment of their togetherness during collective unit 2.

Swimmer	Swimmer’s self-assessment	Expert 1 rating	Expert 2 rating	Expert 3 rating	Expert 4 rating	Expert 5 rating
*S1*	7	5	7	5	6	5
*S2*	4	3	4	4	2	4
*S3*	4	3	4	4	2	4
*S4*	5	3	4	4	2	4
*S5*	1	3	4	4	2	4
*S6*	6	3	4	4	2	4
*S7*	6	3	4	4	2	4
*S8*	7	5	7	5	6	5
*S9*	7	3	4	4	2	4
*S10*	7	3	4	4	2	4
	*54*	*34*	*46*	*42*	*28*	*42*
***Team score***	**54/70**	**38.4/70**				

#### Interviews

**FIGURE 2:**
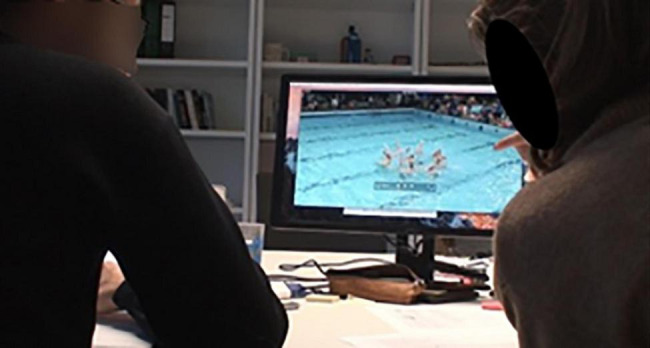
Illustration of the interview situation (the interviewer is on the left and the swimmer is on the right).

Individual interviews were conducted between 1 and 5 days after competition with all swimmers (on average: 3 days) by the first author, who was present during the competition to collect their self-assessments. He confronted the swimmers with the video of their performance, as well as their post-competition self-assessments. He also encouraged each participant to re-enact her pre-reflective experiences that emerged during the competition, helping them to describe her past lived experience (see Legrand, 2006). According to this *second-person* method (see Froese et al., 2011), it is possible to re-enact an experience when one is guided into an appropriate evocation state by a suitably skilled interviewer (see also Hauw, 2009; Vermersch, 2009; Olivares et al., 2015). In this case, the interviewer had experience in conducting similar interviews (see e.g., Gesbert and Durny, 2017; Gesbert et al., 2017; Hauw et al., 2017; Rochat et al., 2018; Gesbert and Hauw, 2020). Each interview was designed as follows: swimmers were first invited to describe, comment on, and explain their behavior for each collective unit of activity by first helping them to rediscover the spatiotemporal context of their past experience (i.e., when, where, with whom, etc.). The first author asked questions that the swimmers could not reply to without referring to the past competition (e.g., “when you were performing this move, what was your main focus?”). The performance video and self-assessments were therefore used to guide and help each swimmer evoke and describe her own experience during this past competition (see Figure 2). To ensure that the swimmers were relating to their own past experience he was attuned to behavioral indicators such as eye shifting or slowing of the word flow, which he considered useful information^1^ (see e.g., Petitmengin, 2006). Second, the swimmers were prompted to describe their experience as it occurred in a specific situation, thus without involving retrospective generalizations or comments.

During the interviews, the first author was also sensitive to verbal indicators (e.g., “at this moment,” “here,” “there”) that the swimmers related to their past experience. Once an evocation of the swimmers’ past experience was established, the interviewer helped them describe this in detail, using questions concerning their associated physical or mental activities (e.g., “what were you doing?”; “what were you thinking about?”), bodily sensations (e.g., “can you describe the main body sensations in this situation?”), concerns and volitions (e.g., “what did you want to do at this moment?”; “what were the main worries during this configuration?”), and elements that were drawing their attention (e.g., “what are you focused on at this moment?”). The interviews lasted between 90 and 120 min each; they were video-recorded and transcribed *verbatim* for further analysis.

#### Experts’ Judgment

Five experts in artistic swimming assessed the togetherness displayed by the swimmers from a *third-person* perspective. As they viewed the performance video, the experts were prompted to judge the level of togetherness displayed by each swimmer for each collective unit of behavioral activity. To do this, they scored togetherness on a Likert scale from (1) “there is no togetherness” to (7) “there is togetherness.” Due to the specificity of the routine investigated (i.e., a routine may be made up of sets of solos, duets, trios and other team segments), the experts were asked to assess this sense of togetherness according to the specific moves performed by the swimmers. As an illustration, if one collective unit of activity was characterized by a group figure (with 8 swimmers) and a duet (see Figure 1b), the experts separately assessed togetherness for the eight swimmers and the two remaining swimmers (see Table 1). By adding these scores together, *a team score* was obtained for each expert. Then, by averaging these team scores, an expert team score was obtained (for which the maximum was also 70).

### Data Analysis

The data analysis consisted of four main phases, each designed to capture a different aspect of the performative experience enacted during the collaborative action. The first three phases corresponded to the analyses of data collected within the three levels described above ( 1st-, 2nd-, and 3rd-person levels). The fourth phase was a joint analysis of these data to reach a more general level of description, as illustrated in detail in the section “Results.” Statistical data analysis was conducted in R (R Core Team, 2020).

#### Assessing Togetherness From Within

First, using the swimmers’ self-assessments about their feeling of being and acting together, each collective unit of behavioral activity was characterized through a team score of being and acting together from the swimmers’ point of view. For instance, collective unit 2 was characterized by a team score of 54 (see Table 1). Thus, by calculating the score of being and acting together from the swimmers’ perspective for each collective unit of activity, the dynamics of this feeling during choreography-performance and the 33 collective units of activity was assessed.

#### Re-enacting Togetherness

**FIGURE 3:**
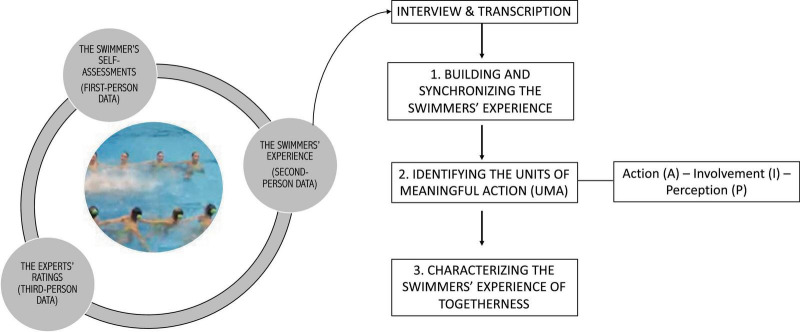
Representation of the three analytic layers, with a special focus on the second-person analytic procedure.

In phase two, the verbal narratives corresponding to the swimmers’ experience were processed in three steps (see Figure 3) following a technique inspired by the *course-of-action* framework^2^ (see e.g., Hauw and Durand, 2008; Hauw, 2009; Poizat et al., 2012; Sève et al., 2013; Mohamed et al., 2015; Theureau, 2015; Rochat et al., 2018). These steps are presented below.

In the first step, the stream of the swimmers’ past experience was rebuilt. To do so, the swimmers’ experiences were progressively connected by presenting them with the collective units of activity in chronological order. This helped them describe their moment-to-moment experiences with accuracy (see e.g., Gesbert et al., 2017). For example, they were able to check on the spot whether their sense of togetherness was convergent during a specific segment of the performance or not.

In the second step, the participants’ transcribed verbal descriptions were categorized as *Units of Meaningful Action* (UMAs) (see e.g., Hauw and Durand, 2008; Sève et al., 2013; Mohamed et al., 2015; Rochat et al., 2018). These UMAs correspond to the smallest units of action that were experienced as meaningful for the swimmer at a given moment. They stemmed from the link between the action and the associated thoughts, or interpretations. UMAs were labeled using a verb followed by a direct object, an adverb, or another complement (e.g., senses that she is unbalanced just before the compression). This coding also helped us simultaneously label the underlying constituents of each UMA, which were identified using a set of more specific questions. Having defined the UMAs, a further categorization differentiated them and described with more precision the range of motor possibilities, perceptive experiences, and proprioceptive feelings that emerged in the choreography. These were labeled as *involvements* (I), *actions* (A), or *perceptions* (P). Involvements were identified by asking the following question: “What were the significant concerns experienced during the choreography?” Actions referred to what the swimmer was actually doing, whereas perceptions included the situations that the participants experienced as significant. As such, P could include the other swimmers’ activity (distance, alignment, compression, etc.), the material environment (e.g., the underwater lights, the pool ceiling etc.), the counts of the choreography and/or the key moments in the music, or a sensation (e.g., bodily contact, balance, etc.). Within each UMA, studying the relationships among I, A, and P helped us capture important aspects of what a person felt, thought, and did (see e.g., Hauw and Durand, 2008; Rochat et al., 2018).

Finally, in the third step, the emergent dynamics of togetherness were explored. The starting point was to describe how each swimmer experienced the feeling of being and acting together in each collective unit of behavioral activity (see e.g., Sève et al., 2013; R’Kiouak et al., 2016; Seifert et al., 2017). This description was based on a detailed examination of two components of each UMA (i.e., I and P). We were thus able to identify different ways of experiencing being and acting together from the swimmers’ perspective.

#### Assessing Togetherness From Outside

The third phase of the analytic process focused on the experts’ assessments. Here, each collective unit of activity was characterized by a team score of togetherness (see Table 1). For instance, collective unit 2 was characterized by a team score of 38.4. This procedure was carried out for all 33 collective units of activity, allowing us to explore the visible dynamics of togetherness from a third-person level as they developed through the performance.

#### A Joint-Methods Approach

In the fourth and final stage of our analysis, we integrated the first-, second-, and third-person data through different combinations to render the dynamics of creativity and togetherness on the artistic swimming team intelligible, and enrich our understanding of the process. The process can be summarized as follows: *first*, by comparing first- and third-person data, we explored whether the swimmers’ feeling of being and acting together corresponded to the togetherness perceived by the experts during performance. *Second*, by scrutinizing the first-person data, we identified the collective units that were felt as problematic by the swimmers. The second-person data were then used to access and expand on the information offered by the swimmers during these specific units. Finally, the third-person data were used to examine how the experts assessed the swimmers’ togetherness in these problematic units. *Third and last*, by examining the second-person data, we first observed how the team (i.e., all the swimmers) experienced a collective feeling of being and acting together during performance. After identifying the collective phenomenological categories for each unit of activity, we focused on specific categories by characterizing them through the first- and third-person data.

## Results and Discussion

In what follows, six main results are presented and contextualized. The first two describe the feeling of being and acting together during the choreography from the swimmers’ points of view. These emerged from the self-assessments (1st person), and from the interviews (2nd person). The third result focuses on the experts’ judgments (3rd person). The last three results emerged from the joint-analysis of the multiple-leveled data to explore in greater detail how changes in individual performance were able to disrupt the unfolding dynamics of interpersonal coordination and how the swimmers were able to compensate for destabilizations in team performance to regain individual and collective stability.

### The Feeling of Being and Acting Together

**FIGURE 4:**
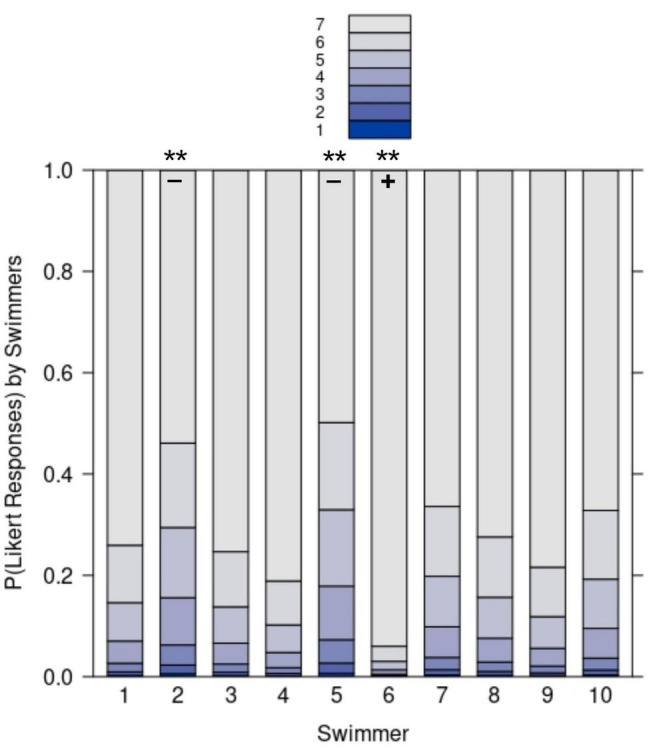
The probability of Likert responses for each swimmer rating their perceived togetherness. Positive (+) and negative (–) deviation of a swimmer from the overall mean are marked if significant (**p < 0.01).

**FIGURE 5:**
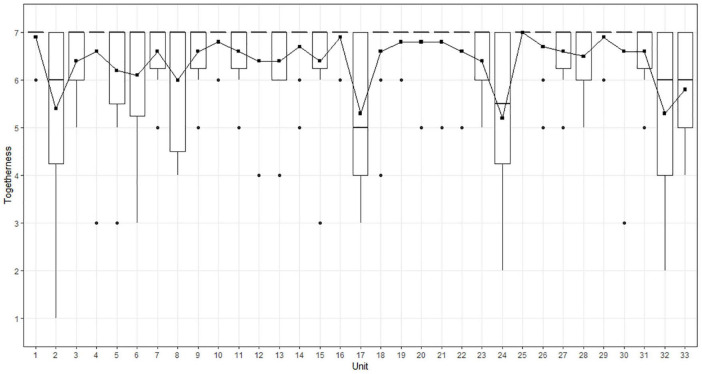
The dynamics of togetherness from the swimmers’ point of view during choreography-performance. The *x*-axis corresponds to the collective units of behavioral activity. The *y*-axis corresponds to the rated togetherness by swimmers. Squares indicate the mean rated togetherness by the swimmers for each unit, which corresponds to the scaled team score by factor 1/10.

The median of the swimmers’ self-assessments for the 33 collective units during the choreography was 7 (IQR = 1). The divergence of each swimmers’ self-assessment from the overall perceived togetherness of the group was quantitatively assessed by calculating an ordinal logistic regression using the polr function from the R package MASS (Venables and Ripley, 2002). To ensure that the parallel regressions assumption was not violated, we ran the brant test (Brant, 1990) using the R package brant (Schlegel and Steenbergen, 2020). In doing so, we determined the statistical model with swimmer as fixed effect factor using sum contrasts (Schad et al., 2020). The analysis showed that self-assessments of swimmer S2 and S5 were significantly lower (S2: *b* = –0.86, *SE* = 0.32, *z*: –2.69, *p*: 0.007; S5: *b* = –1.02, *SE* = 0.33, *z* = –3.13, *p* = 0.002) than the overall swimmers’ self-assessment. On the contrary, self-assessment of swimmer S6 was significantly higher (*b* = 1.73, *SE* = 0.67, *z* = 2.6, *p* = 0.009) than the overall swimmers’ self-assessment. The probabilities for the seven Likert responses according to the model are visualized for each swimmer in Figure 4. So, while S2 and S5 tended to rate togetherness during choreography slightly lower than the other team members and that, in contrast, S6 tended to rate togetherness higher than the others. Figure 5 presents for each collective unit (*x*-axis) the togetherness score at the team level (*y*-axis) corresponding to the addition of the swimmers’ self-assessments of each unit. The curve describes the dynamics of togetherness enacted by the ten swimmers during the performance (as a reminder, the maximum score was 70).

The results showed fluctuations in togetherness as the performance unfolded. Some collective units were characterized by a sudden decrease, such as units 2, 17, 24, and 32, which corresponded to collective moves and/or technical figures for which two or more swimmers felt a weakening in togetherness. This is also indicated by the increased dispersion of ratings for these units as depicted in Figure 5. Yet, these sudden decreases were systematically followed by an immediate or progressive regain (see units 3, 4, 18, or 26). Such a regain of togetherness is also connected in a reduction of dispersion of ratings across consecutive units. To better grasp these team scores, we scrutinized the swimmers’ individual perspectives (see Table 1). For instance, for unit 2, the sudden decrease at the team level may be explained by the perspective of six swimmers (S2, S3, S4, S5, S6, and S7) who were performing a highlight (see Figure 1) and felt a weakening in their togetherness during this figure. Then, to better grasp the swimmers’ weakening in their togetherness, we explored how they described their lived experience during this specific figure.

### Swimmers’ Experience Rebuilt

The analysis performed on the swimmers’ interview data indicated four ways of experiencing togetherness (see Table 2). The 330 UMAs corresponding to the ten swimmers’ experience during the 33 collective units were examined.

**TABLE 2:** The swimmers’ experience of togetherness during choreography-performance.

	Perceptions (P)	Involvements (I)
Togetherness (T)	The habitual bodily contact with another swimmer The right tempo with other swimmers The nice “shape/form” of the formation The right alignment with and right distance from the other swimmers The timing of the movements	Maintain the right distance and stay in contact with the other swimmers Focus on being aligned with the other swimmers
Weakened Togetherness (WT)	Not sufficiently aligned A little too close to or too far from the other swimmers A little too close to or too far from a partner Insufficient compression	Adjust in order to be aligned Slow down or speed up one’s movements Push the partner to be in her place Put in a little more effort Reduced time for adjusting self
Absence of Togetherness (AT)	A swimmer has lost the count Body contact is much more condensed than usual The formation has no shape The sensation of being pushed diagonally Chaos while getting into place underwater	Is unable to adjust Has no say in the adjustment process Is prevented from adjusting Has no time to adjust
Meaningless Togetherness (MT)	Each has her own count and her own sensations The coach’s instructions	Be as aesthetically pleasing as possible Be tuned into one’s own sensations Recall the coach’s individual instructions

The first dimension was the *experience of togetherness* (T), corresponding to 71% of the UMAs (236/330). It was characterized by the swimmers’ feeling of effectively interacting with the others and producing the choreographic performance that was expected. As an example, consider the following quote:

*“There, we just did the body boost and then it’s the start of the lift. As I’m the swimmer who’s the farthest away, I have priority to pass. I do a long breaststroke and I feel that the girls let me through. I thought it was very good, this transition is going well, I don’t need to put in extra effort.” (S6, Collective Unit 24).*

The second category that emerged from the second-person data was the *experience of weakened togetherness* (WT), corresponding to 14% of the UMAs (46/330). It was characterized by the feeling of not being sufficiently coordinated to produce the expected choreographic performance. The following two quotes exemplify this feeling:

*“S3 is in front of me, I put my hand on her shoulder to check for the correct distance between us and I have to check the alignment with S10, who’s in the other line. I see that we’re not too aligned with S10, so I tried to slow down to get into alignment with her.” (S9, Collective Unit A7).*

*“On this mini-lift, I’m with S3 but it doesn’t help much. This lift is really hard. I feel it isn’t high enough out of the water*… *Yeah, S3 isn’t helping me enough.” (S10, Collective Unit 28).*

The third category was the *experience of the absence of togetherness* (NT), corresponding to 10% of the UMAs (33/330). This was expressed as bad feelings about collective action, as if something was wrong or was not happening as usual. Consider the following quotes from two swimmers:

*“Before I jumped, I felt like it wasn’t going to work. I’m watching S2 and S7 so I can get on top of them, but I couldn’t see their hands but didn’t know why! I’m way behind,… here I’m completely behind.” (S5, Collective Unit 2).*

*“I move to the left to be next to S6. The others carry us from behind. I feel the pressure of feet below S7, and the pressure of S5’s feet above me. Here we’re way too early. When I started to build power, I felt S5 leaving, although for me it was not at all on the count. Usually, I feel that she has time to position her feet and there the moment was very condensed compared to the usual.” (S2, Collective Unit 2).*

Finally, the last category that emerged from the interview was the *experience of meaningless togetherness* (MT), for 5% of the UMAs (15/330), especially noted when the swimmers performed technical figures: with the upper body, with the legs (e.g., ballet leg), with spins or solo. This experience could be defined as the feeling of being attuned only to one’s own movements, rather than those of the rest of the team. Relevant examples can be found in the following verbal descriptions:

*“There I have to do a body boost with S2, but I’m only tuned in to my own boost because I can’t see S2, who’s behind me.” (S5, Collective Unit 24).*

*“Here, I’m paying attention to make the small corrections that the coach gave me and I’m only tuned in to my own figure, looking for my own bodily sensations. The angle is super important, I can’t let my legs be too low or too high*… *Once I feel that I have it (the right height), I keep it here.” (S8, Collective Unit 30).*

### Perceiving Togetherness

**FIGURE 6:**
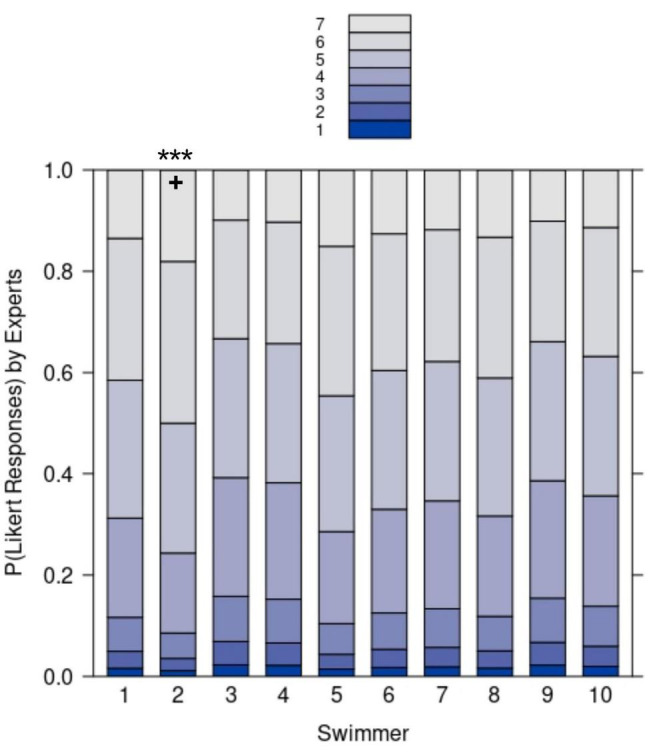
The probability of Likert responses of experts rating the togetherness of each swimmer. Positive (+) deviation of a swimmer from the overall mean are marked if significant (***p < 0.001).

**FIGURE 7:**
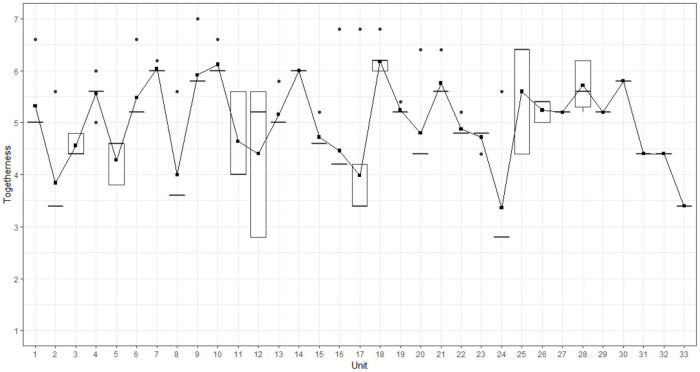
The dynamics of togetherness from the experts’ point of view during choreography-performance, rated for each swimmer. The *x*-axis corresponds to the units of behavioral activity. The *y*-axis corresponds to the rated togetherness by experts. Squares indicate the mean rated togetherness by experts for each unit, which corresponds to the scaled team score by factor 1/10.

The median of the experts’ ratings for the swimmers’ togetherness for the 33 collective units was 5 (IQR = 2). We assessed the interrater reliability by calculating the intraclass correlation coefficients (ICC) as described by Koo and Li (2016). ICC estimates and the 95% confidence intervals were calculated with the help of the R package “psych” (Revelle, 2021) on base of a mean rating (k = 5), absolute agreement, two-way mixed effect model. We report a moderate to good ICC for the expert ratings (ICC = 0.73, 95% CI [0.68, 0.78]). As with the analysis of swimmers’ self-assessment, we aimed at exploring which swimmers deviated from the group as assessed by the expert ratings. To do so, an ordinal logistic regression was performed using the polr function from the R package Mass (Venables and Ripley, 2002). The model was set up using sum contrasts (Schad et al., 2020) and swimmer as fixed effect factor. Results showed that experts rated only the togetherness of swimmer S2 as significantly higher (*b* = 0.44, *SE* = 0.13, *z* = 3.3, *p* < 0.001) compared to the overall rated togetherness of the group. The probabilities for the seven Likert responses according to the model are visualized for each swimmer in Figure 6. Figure 7 depicts the mean togetherness score for each collective unit (*x*-axis) from the experts’ point of view (*y*-axis). The curve represents the dynamics of togetherness at the team level assessed by the experts during the choreography-performance (as a reminder, the maximum score was 70).

Some units were characterized by a sudden decrease, such as units 2, 5, 8, or 24 (corresponding to discrete movements), whereas units 15, 16, and 17 (corresponding to a set of specific and linked movements during the choreography) were characterized by a progressive decrease. To better grasp these fluctuations, we scrutinized the experts’ individual perspectives, which revealed how each expert rated togetherness for each swimmer (see Table 1). For instance, for unit 2, all the experts assessed the togetherness of the swimmers performing the highlight (i.e., S2, S3, S4, S5, S6, S7, S9, and S10) between 2 and 4 on the 7-point Likert scale, whereas togetherness for the swimmers involved in a duet (S1 and S8) was assessed between 5 and 7.

### Joint Analysis Between First-, Third-, and Second-Person Data

**FIGURE 8:**
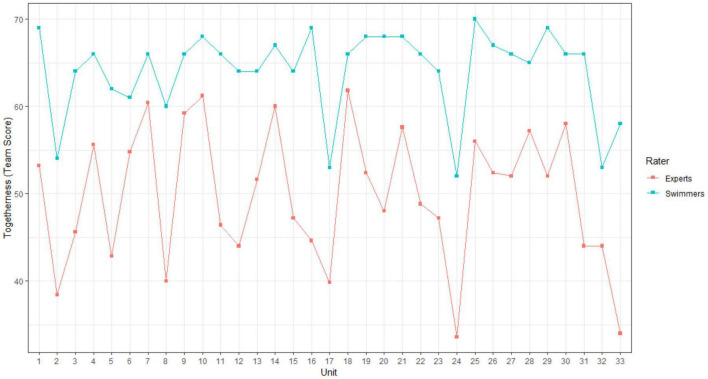
Joint analysis between first- and third-person data. The blue curve above corresponds to the team score of the swimmers’ self-assessments (first-person data). The curve below corresponds to the team score of the experts’ ratings (third-person data).

For this procedure, we first scrutinized the comparisons between the first- and third-person data in order to delineate the samples of the second-person data to be analyzed. The first objective was to understand how the swimmers’ experience of being and acting together fit with the togetherness assessed by the experts (see Figure 8). Hence, we ran an ordinal logistic regression using the polr function from the R package Mass (Venables and Ripley, 2002) to compare the ratings of both groups across all units. The model was set up using sum contrasts (Schad et al., 2020) and rater group as fixed effect factor. This comparison revealed that, on average, swimmers rated the perceived togetherness significantly higher (*b* = 1.3, *SE* = 0.07, *z* = 18.93, *p* < 0.001) than experts. This coherent deviation of swimmers and expert ratings can also be found when inspecting the summed ratings for each unit of the choreography in Figure 8 (i.e., the orange curve was always below the blue curve). The figure shows that the swimmers’ feeling of acting together is always exceeded by the experts’ perceptions of togetherness for all the units of activity. Three most and least diverging units were identified: units 16, 33, and 31 and units 6, 18, and 7 respectively (in increasing order of divergence).

Overall, the shapes of the curves present the same profile with ascendant and descendant trajectories, except for eight transitions. For instance, between units 18 and 19, the swimmers’ feeling of acting together increased, whereas the assessment of togetherness from the experts’ perspective decreased. In contrast, between units 29 and 30, the swimmers’ feeling of acting together decreased, whereas the assessment of acting together from the experts’ point of view increased. When identifying the collective units for which the swimmers’ feeling of acting together decreased whereas the assessment of togetherness from the experts’ point of view increased (i.e., transitions between units 5–6, 27–28, 29–30, and 31–32), we scrutinized the swimmers’ experience from their point of view (see Table 3).

**TABLE 3:** The swimmers’ experience during collective unit 32.

Swimmers	The feeling of being and acting with each other	Unit of meaningful action (UMA)	Involvement (I)	Perception (P)
S1	7	Backs up to be in the circle	To participate in forming a circle with the other swimmers	Aligned with the swimmer opposite her

S2	2	Tells self that they’re too spread out just before turning around	To be unable to adjust	Sees swimmers on the side she’s on that she shouldn’t be able to see

S3	5	Realizes that they’re not at all together when she turns around	To be unable to adjust	Swimmers next to her

S4	7	Has the impression that all is correctly positioned behind her	To line up to be in the circle	The positions of S7 and S10
	
		Realizes that it’s not the case when she turns	To turn around to perform the figure	The swimmers’ positions

S5	4	Turns around and perceives that they are too far apart to be in formation	To look at the swimmer at the end and be at the right distance from the nearby swimmers	Alignment and distance

S6	7	Backs up to be in the circle	To try to line up and be at the right distance	The alignment in a square

S7	3	Backs up to be in the circle	To be aligned with S2 and the right distance from the nearby swimmers	Alignment and distance with the swimmers
	
		Realizes that they’re not in a circle when she turns around	To be unable to adjust	Poor alignments

S8	7	Gets adjusted with her partner	To adjust with S1	S1

S9	7	Gets a good feeling about the figure being performed	To line up with the opposite swimmer and manage the distance with the other two swimmers	Alignment and distance with the swimmers

S10	4	Backs up to be in formation	To get between S5 and S8 and stay attentive to the alignment with S3	Positions of S3, S5 and S8
	
		Turns and sees the catastrophe	To be unable to adjust	Poor alignments

For the transition between units 31 and 32, togetherness slightly increased from the experts’ point of view (i.e., the team score varied between 40 and 44), whereas the swimmers felt a substantial weakening in their feeling of being and acting together (i.e., the team score varied between 66 and 54). The second-person data indicated that most of the swimmers (i.e., S3, S4, S5, S7, and S10) perceived that the shape being enacted by the set of swimmers did not correspond to what it was expected only after they had turned around. Only then did they realize that they were in trouble not so much in terms of alignment as in distance and that they were now unable to cope. Only one swimmer (S2) explained that, just before turning around, she had realized that they were not together because she could see the swimmers to the side, which she could not usually do when the figure was performed correctly, though she could see no possibility of adjustment.

### Joint Analysis Between First-, Second-, and Third-Person Data

**FIGURE 9:**
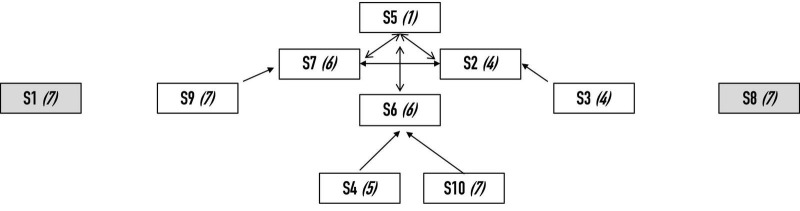
The individual swimmers’ perceptions of the feeling of being and acting together for collective unit 2.

This procedure involved first scrutinizing the first-person data to delineate the samples of second-person data to be analyzed. From the first-person data (see Figure 7), we were able to identify all the units that had been experienced as problematic, with the team score of feeling of acting together below 60 (i.e., units 2, 3, 6, 8, 17, 24, 32, and 33). Although this value of 60 was subjective, it indicated that two or more swimmers felt a weakening in their feeling of being and acting together. For instance, unit 2 was characterized by a team score of 53. The individual swimmers’ perceptions of feeling of acting together for this unit are indicated in brackets in Figure 9.

The number in parentheses indicates the score for feeling of being and acting together expressed by each swimmer from her perspective.

Six swimmers felt a weakening in their feeling of being and acting together (i.e., S2, S3, S4, S5, S6 and S7), and these six swimmers were involved in performing a specific figure (i.e., a highlight^3^). We investigated their experience to determine what was meaningful for them at this instant in the situation (see Table 3). The results indicated the rich variety of information that the swimmers drew on to explore their feelings of being and acting together. Included were the alignment with one or more swimmers, the push with one or more swimmers, the expected position of one swimmer, the compression co-built with another swimmer, the balance built with other swimmers, and the time taken to achieve a shape. In the last stage, the five experts’ assessments of their togetherness were examined to determine how they fit or did not fit with the swimmers’ feelings of being and acting together (see Table 4). These third-person data offered the opportunity to observe whether the experts were attuned to the big problems (i.e., characterized through a low team score of togetherness) or the global form enacted by the swimmers. In this last case, low variability was acceptable for the experts.

**TABLE 4:** Contribution from first-, second- and third-person data for unit 2.

Swimmers	Feeling of being and acting together	Perception (P)	Expert 1′s ratings	Expert 2′s ratings	Expert 3′s ratings	Expert 4′s ratings	Expert 5′s ratings
S1	7	Alignment with another swimmer	5	7	5	6	5
S2	4	S5**′**s push was more condensed than usual	3	4	4	2	4
S3	4	A setback in the boost phase, more difficult for her to push S2	3	4	4	2	4
S4	5	The diagonal position of S6 (rather than the expected vertical)	3	4	4	2	4
S5	1	Feels too far back in the platform (imbalance)	3	4	4	2	4
S6	6	Sensitive to body contact with S2 and S7, then with S3 – some difficulty moving and orienting/localizing herself	3	4	4	2	4
S7	6	The time S2 needed to find her and S6	3	4	4	2	4
S8	7	Alignment with another swimmer	5	7	5	6	5
S9	7	Bodily contact with S7 and perception of S5**′**s difficulty in finding S2 and S7	3	4	4	2	4
S10	7	The feeling of pushing with S3 and S6	3	4	4	2	4

### Joint Analysis of the Second-, First-, and Third-Person Data

In this procedure, we first scrutinized the second-person data to delineate the samples of the first- and third-person data to be examined. As a reminder, the analysis of the swimmers’ interview data revealed four ways of experiencing togetherness (see Table 2). *Meaningless togetherness* was used to indicate that the swimmers were not paying attention to togetherness at the pre-reflective level of their activity (i.e., labeled MT, in purple). The other types of experience accounted for the UMAs in which the swimmers reported salient experiences of togetherness. The *absence of togetherness* accounted for the UMAs in which the swimmers reported that they were not being and acting together (labeled AT, in red). Weakened togetherness accounted for the UMAs in which the swimmers reported a meaningful experience of a weakening in being and acting together (i.e., labeled WT, in green). *Togetherness* (T) accounted for the EUMs in which the swimmers reported a meaningful experience of being and acting together (in yellow). For each collective unit of behavioral activity, the experience of each swimmer was labeled in one of these four phenomenological categories in relation with their position on the team (see Table 5).

**TABLE 5:** The swimmers’ experience of togetherness during choreography-performance.

*UNIT 1*	*PLATFORM LIFT*								*DUET S1 and S8*	
	T	T	T	T	T	T	T	T	T	T
*UNIT 2*	*HIGHLIGHT*								*DUET S1 and S8*	
	T	WT	WT	WT	AT	AT	AT	AT	T	T
*UNIT 3*	*BODY BOOST*						*MINI LIFT*			
	T	T	T	T	T	WT	AT	AT	T	WT
*UNIT 4*	*HIGHLIGHT*						*FIGURE S7 and S3*		*S2 and S6*	
	T	T	T	T	T	T	T	T	T	AT
*UNIT 5*	*BODY BOOST*						*S7 and S3 – S2 and S6*			
	T	T	WT	WT	WT	MT	T	T	T	T
*UNIT 6*	*HIGHLIGHT*								*DUET S9 and S10*	
	T	T	T	T	WT	WT	AT	AT	T	T
*UNIT 7*	*MOVE IN TWO LINES*								*DUET M* + *B*	
	T	T	T	T	T	T	WT	WT	T	WT
*UNIT 8*	*TWO-WAVE LIFT*								*MINI-LIFT*	
	T	T	T	T	AT	AT	AT	AT	T	T
*UNIT 9*	*FIGURE*									*SOLO*
	T	T	T	T	T	T	WT	WT	WT	T
*UNIT 10*	*CIRCLE*								*LIFT S1 and S6*	
	T	T	T	T	T	T	WT	WT	T	T
*UNIT 11*	*MOVE WITH LEGS*						*MOVE WITH ARMS*			
	T	T	T	T	WT	MT	T	T	T	WT
*UNIT 12*	*MINI-LIFT*				*MINI-LIFT*				*BODY BOOST*	
	T	T	T	T	T	T	T	T	AT	MT
*UNIT 13*	*FIGURE*								*DUET*	
	T	T	T	T	T	T	WT	WT	T	WT
*UNIT 14*	*FIGURE WITH ARMS*								*DUET*	
	T	T	T	T	T	T	T	W	T	M
*UNIT 15*	*TEAM* + *SPIN*								*DUET*	
	T	T	T	T	WT	AT	MT	MT	T	T
*UNIT 16*	*SURFACE FIGURE*									*SOLO*
	T	T	T	T	T	T	T	T	T	MT
*UNIT 17*	*BALLET LEGS*						*BODY BOOST*			*SOLO*
	AT	AT	MT	MT	MT	MT	T	T	WT	MT
*UNIT 18*	*LIFT S5 (FIRST BASIS)*					*LIFT S5 (SECOND BASIS)*				*SOLO*
	T	T	T	T	WT	T	T	T	T	T
*UNIT 19*	*FIGURE WITH ARMS*								*DUET*	
	T	T	T	T	T	T	WT	WT	T	T
*UNIT 20*	*BOX-BOX FIGURE* + *2 LINES*								*DUET*	
	T	T	T	T	T	T	T	WT	T	T
*UNIT 21*	*ONE-LINE FIGURE*								*DUET*	
	T	T	T	T	T	T	T	WT	T	T
*UNIT 22*	*BODY BOOST BY 2*								*DUET*	
	T	T	T	T	T	T	T	WT	T	WT
*UNIT 23*	*TWO LINES*								*DUET*	
	T	T	T	T	T	T	T	WT	WT	WT
*UNIT 24*	*LIFT S3*								*BODY BOOST*	
	T	T	WT	WT	WT	AT	AT	AT	T	MT
*UNIT 25*	*BODY BOOST*				*UNDERWATER MOVE*				*BARRACUDA*	
	T	T	T	T	T	T	T	T	T	T
*UNIT 26*	*MOVE*				*MOVE (KICK)*					
	T	T	T	T	T	T	T	T	T	WT
*UNIT 27*	*FIGURE*									
	T	T	T	T	T	T	T	T	WT	A
*UNIT 28*	*BODY BOOST*			*MINI-LIFT*				*BODY BOOST*		
	T	T	AT	T	T	T	AT	T	T	T
*UNIT 29*	*FIGURE WITH THE UPPER BODY*									
	T	T	T	T	T	T	T	T	T	T
*UNIT 30*	*BOX-BOX FIGURE*									
	T	T	T	T	T	T	T	T	MT	MT
*UNIT 31*	*FIGURE WITH TWO LINES*									
	T	T	T	T	T	T	T	T	WT	AT
*UNIT 32*	*FIGURE IN CIRCLE* + *BODY BOOST CIRCLE*									
	T	T	T	WT	AT	AT	AT	AT	AT	AT
*UNIT 33*	*LIFT S5*									
	T	T	T	T	WT	WT	WT	AT	AT	AT

*T, Togetherness; WT, Weakening Togetherness; AT, Absence of Togetherness; MT, Meaningless togetherness.*

Four collective phenomenological categories were identified:

- CPC1: Simultaneously and Similarly Experienced as Togetherness at team level.
- CPC2: Simultaneously and Similarly Experienced as Togetherness within a subgroup and simultaneously diverging experience in another subgroup.
- CPC3: Simultaneously Diverging Experiences (i.e., two different ways of experiencing togetherness within two subgroups).
- CPC4: Simultaneously Highly Diverging Experiences (i.e., three or four ways of experiencing togetherness at the team level or within two subgroups).

These categories helped us grasp whether the swimmers similarly or differently perceived their being and acting together. Then, for each collective unit, the average individual scores for the feeling of being and acting together (first-person data) and the perception of togetherness (third-person data) were indicated for each move or figure characterizing this collective unit. For instance, units 1, 2, and 3 were characterized by two distinct moves/figures, whereas unit 12 was characterized by three distinct moves.

For each of these moves, the results indicated the average individual score for the swimmers and the experts, as well as the standard deviation (see Table 6).

**TABLE 6:** Contribution from second-, first-, and Third-person data.

Collective units	CPC	Swimmers’ feeling of togetherness	Experts’ perceptions of togetherness	Swimmers’ feeling of togetherness	Experts’ perceptions of togetherness	Swimmers’ feeling of togetherness	Experts’ perceptions of togetherness
1	CPC1	6.9 *(0.3)*	5.53 *(0.9)*	7 *(0)*	6.6 *(0.5)*		
2	CPC4	5 *(2)*	3.4 *(0.8)*	7 *(0)*	5.6 *(0.8)*		
3	CPC4	6.33 *(1)*	4.4 *(1.4)*	6.5 *(0.6)*	4,8 *(1,4)*		
….	…	….	…	….	…	….	…
12	CPC4	7 *(0)*	5.6 *(1.2)*	7 *(0)*	2.8 *(1.4)*	4 *(0)*	5.2 *(1)*
…	…	….	…	….	…	….	…
32	CPC4	5.3 *(1.9)*	4.4 *(0.5)*				
33	CPC4	5.8 *(1.2)*	3.4 *(0.5)*				

*CPC: Collective Phenomenological Categories; CPC1: Simultaneously and Similarly Experienced as Togetherness; CPC2: Simultaneously and Similarly Experienced as Togetherness within a subgroup and simultaneously diverging experience in another subgroup; CPC3: Simultaneously Diverging Experiences in Two subgroups; CPC4: Simultaneously Highly Diverging Experiences.*

For collective unit 12, for example, the results showed that CPC4 was characterized from the swimmers’ perspective through a single move between two swimmers (i.e., subgroup 3) in which togetherness was rated 4. However, the experts perceived togetherness during this move as higher than the swimmers’ perceptions (Msubgroup 3 = 5.2). In contrast, for other moves in which the swimmers’ perceived togetherness was equal to 7, the mean of the experts’ ratings was lower (Msubgroup 1 = 5.6; Msubgroup 2 = 2.8).

## General Discussion and Conclusion

The aim of this paper was to offer a detailed description of the feeling of being and acting together in the context of collaborative artistic performance. The feeling of being and acting together has often been understood as a crucial dimension for optimal collaborative activity in sports and music (see e.g., Lund et al., 2014; Schiavio and Høffding, 2015; Himberg et al., 2018). We therefore chose to focus on synchronized swimming as it requires skill in both aesthetic and athletic components (e.g., rhythmical entrainment, competitiveness, sportsmanship, etc.). We first developed two assessment instruments so that the swimmers could evaluate their feeling of being and acting together and the expert raters could also evaluate their togetherness, thus providing us with first- and third-person perspectives. We then conducted interviews based on elicitation techniques in order to perform a second-person level of analysis (see e.g., Gesbert et al., 2017; Gesbert and Hauw, 2020). This allowed us to explore in greater detail the moment-to-moment experiences that permeated the swimmers’ activities at given moments. By combining these methodological approaches *via* joint analysis, we obtained precise descriptions of how the changes in individual and collective behavior shaped, disrupted, and re-stabilized an artistic performance.

We found that the swimmers who took part in the study were highly attuned to their feeling of being and acting together during the execution of the choreography. Although this result was not fully surprising, the combination of multiple analytical tools helped us provide a detailed description of the interplay between the singular and plural dynamics at the heart of team effort. By integrating the scores of togetherness assigned to each unit of behavioral activity with the verbal descriptions from the interviews, the fluctuating, situated nature of the feeling of being and acting together emerged. In particular, our analysis suggests that despite the planned patterns of behavior defining the choreography, this latter is less static than one might think. Indeed, artistic swimmers often adjust their behaviors in light of an immediate experience of interaction with one or many team members: our first-person data (self-assessments) revealed how they individually feel togetherness during competition. Since they act and react according to a set of dynamical and evolving constraints (see Davids et al., 2007), their experience of togetherness is transient and constantly oscillating between increases and decreases of felt togetherness.

These first-person data also showed how, after each marked decrease in togetherness at the group level, an immediate active response occurred. The feeling of being and acting together therefore appeared to be crucial to creatively engaging with the contingencies and perturbations of performance, allowing the swimmers to immediately and efficiently adapt to their teammates and their situated activity. Even though the swimmers sought to actively regulate the interaction process as they accomplished a figure or a move together, they were constrained by ecological information: according to their position and their role in the choreography, they experienced different feelings of togetherness. This may explain why, during a highlight, the flyer rated togetherness as a 1 on the 7-point Likert scale, whereas the other swimmers rated it a 7. The flyer, who was situated at the top of the platform lift, felt the set of compressions produced by her teammates, whereas the other swimmers could only feel more attenuated parts of the compressions. It should be noted that the aquatic environment is inauspicious for exchanging information, and the choreography lasts 4 min with a preestablished chaining of figures, highlights, and moves. As such, it is fundamental to establish alternative ways of accessing information related to the behavioral dynamics of others, thereby creating a synergetic “we-experience” that transforms individuality and collectivity on the basis of a subtle sense of togetherness. In artistic swimming, togetherness is bonded in the very tuning of the performers’ interactions with others as they strive for accuracy in the lines and positions of the formation, the distance they maintain between themselves, the rate at which the formation shifts, the beauty or aesthetic of the emerging figure, the tempo of their figures, and the musical interpretation expressed *via* their synchronized movements to the music.

Another important outcome of our analysis concerned the four ways in which artistic swimmers experience the sense of togetherness. We labeled these ways as: *togetherness*, *weakened* togetherness, *absence* of togetherness and *meaningless* togetherness. Each of these experiences arguably emerges from the integration of audio-visual and proprioceptive information about the alignment and/or the distance from other swimmers, the position within the formation, and the building of balance, timing and movements in relation with others—or rather their own feelings of staying synchronized with the others (see e.g., Gesbert and Hauw, 2020; Toner and Montero, 2020). In this last case, although a small part of the coded UMAs were characterized as meaningless togetherness, all of them were associated with specific segments of the team performance, thus revealing the situatedness of this experience during the choreography. By engaging with such information, the swimmers were able to adjust their activity and perform together (see I and UMA). Yet, however rich this information may have been, it could not always provide them with all the necessary resources to engage in the complex dynamics of their performance. Again, adaptations need to be made immediately, sometimes with enormous risks for the collective performative outcome. Accordingly, the swimmers also relied on a complementary set of tools centered on a more conative dimension (see Legrand, 2006). This also explains the rich variety of feelings associated with the choreography, which often fluctuated between the concrete immediacy of their activity and the expected outcome that was collectively built through hours of collective practice prior to the performance. Indeed, the swimmers’ experience of full togetherness was often hampered by difficulties. These perceived “difficulties” were directly due to the structure of the choreography (i.e., the preestablished chaining between some of the moves was too difficult for some of the swimmers) or the swimmers’ activity, such as inadequate positions, insufficient and/or unsatisfactory movements and so on. The interactions affecting their collective and individual activity were analyzed through the interviews, in which the swimmers were prompted to describe, comment on and explain the differences between the ideal and unfavorable conditions of reciprocal interaction and co-regulation.

The analysis of the expert ratings confirmed the key role of togetherness in determining how the various interpersonal synergies unfolded, with a special emphasis on the individual level rather than group level. Unlike other studies that have sought to characterize team performance in artistic and sports contexts (Sève et al., 2013; Vicary et al., 2017; Himberg et al., 2018), the present study dealt with artistic swimming, where a choreography can often be split into two or three interdependent subgroups. Within each subgroup, the performers seek to efficiently adjust their activity to the needs of the collective activity, such as, for instance, sufficiently pushing another swimmer upward before a highlight and adjusting the rate of their leg movements or their position within the formation in order to promote synchronized movements or better alignment among the swimmers. In the present study, only six of the 33 collective units were rated at the team level. For the other collective units, the experts were more attuned to the interaction process between two or more swimmers involved in the same move or figure (or in the achievement of the same task).

One of the main advantages of the joint-methods approach is the mutual enrichment of the domains of evidence, such as is offered by first-, second-, and third-person perspectives. The main idea is that putting together several levels of analysis can generate novel insights that recursively enrich each other, thereby bringing the subtle nuances of intersubjectivity into the daylight of lived experience (see Depraz et al., 2017, p. 192). A good example of this emerged from the integration of first- and third-person data, which permitted us to analyze two main aspects of the swimmers’ performance. The first aspect was the coherent discrepancy emerging from the direct comparison of the swimmers’ and raters’ assessments: across all units, experts rated togetherness 1.3 points lower than swimmers. The second aspect, which the elision of the first and third levels of analysis brought forth, provided a description of the three most and least diverging units of behavioral activity (units 6, 18, and 7 and units 16, 33, and 31 respectively) between swimmers’ and experts assessments. This showed that the swimmers’ and experts’ perspectives on these specific collective units differed from the otherwise coherent divergence between the group ratings. To account for these differences, the constitutive elements of the swimmers’ experience (UMAs, I, P) in these six collective units were given special attention during the interviews. Doing so provided us with new understandings of the swimmers’ experience of togetherness as we explored in detail whether they adjusted (and how) their behavior in these specifically problematic moments and thereby enriched the initial data. Combining the first- and third-person data was not enough to yield precise insights into what information was meaningful for the team members to rate togetherness. During the individual interviews, all the performers were thus confronted with their togetherness self-ratings, and invited to comment on, explain, and describe in detail what they had felt during the performance.

This analytic approach builds on and extends the literature by providing an apt counterpoint to studies that focus separately on qualitative and quantitative data. For instance, there is a vast literature in the sports sciences that presents phenomenological data (i.e., second-person data) to identify the relevant dependent variables to be explored quantitatively (see Sève et al., 2013; R’Kiouak et al., 2016; Rochat et al., 2019). In a similar vein, other scholars often focus on behavioral data (i.e., third-person data) and then enrich these data with verbal accounts by the participants (see e.g., Seifert et al., 2017). By adding a further level of analysis, the present contribution provides a more holistic, real-time description of how togetherness evolves and shapes performance. Given the specificity of artistic swimming and the importance of its aesthetic dimension, we used expert perspectives as third-person data. Contrary to other studies in sports that have used biomechanical or behavioral indicators to assess, for instance, the “synchronization of the rowers” (see Sève et al., 2013; R’Kiouak et al., 2016; Seifert et al., 2017), in the present study we asked experts to rate how they perceived the swimmers being and acting together on a 7-point Likert scale. However, it might also be possible to look for behavioral indicators in the swimmers’ experience that would account for the togetherness they feel. For instance, future studies could measure the distance and/or alignment between the swimmers at the most difficult moments of the choreography and compare these data with the subjective accounts emerging from the first and second levels of analysis. This can also include a broader examination of the target population’s creative potential. Consequently (at least in the contexts of creative togetherness described in this work), not only the joint methods approach has a precious ally in the theoretical resources of works on dynamic systems and ecological dynamics (see e.g., Araújo and Davids, 2004; Araújo et al., 2006; Araújo et al., 2017; Kimmel, 2017, 2019; Schiavio and Kimmel, 2021); it could also be integrated with specific tests and measurements relating to individual and group (motor) creativity (see e.g., Bournelli et al., 2009; Grammatikopoulos et al., 2012; Santos and Monteiro, 2021) in order to increase its explanatory power. This might be beneficial when it allows for broader analytical tools combining interactions at the micro and macro level, the experience and formation of meaningful behavioral patterns, and the creative drive that favors different types of exploration and problem-solving dynamics at multiple scales (see also Hristovski et al., 2012; Orth et al., 2017).

Before concluding, we should note again that the context of artistic swimming may limit generalization, although certain collective activities (such as competitive dance performances^4^ for instance) may display similar features. The team performance was segmented into discrete collective units of behavioral activity to both structure the individual interviews and facilitate the comparison between the swimmers’ pre-reflective experiences of togetherness during the choreography. Compared with other studies in a sports or artistic context, our study of team performance in artistic swimming relied on the interdependent and independent contributions of the swimmers: interdependent in the sense that the swimmers performing the same task had to act and react together, and independent in the sense that their contributions may also have been focused on performing specific subtasks that involved micro-processes of self-other adaptation. Moreover, unlike other sports contexts in which the use of biomechanical or behavioral indicators has been established in training and performance analysis, this is not the case in artistic swimming, and we therefore focused on other parameters. In conclusion, although the research to date has described being and acting together as an essential aspect of team performance, the present contribution is the first to offer a comprehensive analysis based on joint methods. Our study suggests that the feeling of togetherness experienced by a team of swimmers during a choreography is constantly mutating and is both task-specific and task-general. Interestingly, the continuous increases and decreases of sense of togetherness reported by our swimmers can be consistently recognized by expert raters. And indeed, except for eight transitions, the rated togetherness by experts follows closely the same trajectory of the togetherness emerging from the swimmers’ ratings. They both fluctuate in a very limited range of togetherness. We hope future research will engage with similar considerations and extend the analysis to other domains in sports and artistic performance.

## Data Availability Statement

The anonymized raw data supporting the conclusions of this article will be made available by the authors, without undue reservation.

## Ethics Statement

Ethical review and approval was not required for the study on human participants in accordance with the local legislation and institutional requirements. Written informed consent to participate in this study was provided by the participants’ legal guardian/next of kin.

## Author Contributions

VG designed the study, collected the data, analyzed the data, and wrote and edited the manuscript. DH designed the study and edited the manuscript. AK analyzed the data and edited the manuscript. AB edited the manuscript. AS wrote and edited the manuscript. All authors contributed to the article and approved the submitted version.

## Conflict of Interest

VG was employed by Football Club Lorient. The remaining authors declare that the research was conducted in the absence of any commercial or financial relationships that could be construed as a potential conflict of interest.

## Publisher’s Note

All claims expressed in this article are solely those of the authors and do not necessarily represent those of their affiliated organizations, or those of the publisher, the editors and the reviewers. Any product that may be evaluated in this article, or claim that may be made by its manufacturer, is not guaranteed or endorsed by the publisher.

## Footnotes

1 These behavioral indicators were inserted into the interview transcripts. This allowed us to remove elements (such as retrospective generalizations or comments) which we considered had no direct relationship to the swimmer’s experience during the competition.

2 As a methodological framework, the *course-of-action* technique (see Theureau, 2015) aims to help athletes rebuild their activity by re-enacting their experience (i.e., what they perceived, felt, and did at a given moment).

3 This technical term in artistic swimming refers to an acrobatic movement with a platform of swimmers who unite their maximal power to propel one of them, the flyer (in this case, S5), completely out of the water.

4 We thank one of our reviewers for suggesting this link.
